# Psychometric properties of the PTSD Checklist for DSM-5: a pilot study

**DOI:** 10.3402/ejpt.v7.30165

**Published:** 2016-04-19

**Authors:** Josefin Sveen, Kristina Bondjers, Mimmie Willebrand

**Affiliations:** Department of Neuroscience (Psychiatry), Uppsala University, Uppsala, Sweden

**Keywords:** PTSD, PCL-5, child burn, parents, physical trauma, validation

## Abstract

**Background:**

To date there is a lack of studies assessing the psychometric properties of the recently revised PTSD Checklist (PCL), the PTSD Checklist for DSM-5 (PCL-5). The aim of this pilot study was to examine the psychometric properties of the PCL-5 in parents of children with burns.

**Methods:**

The participating parents (*N=*62, mean age=38) completed self-report questionnaires, 0.8–5.6 years after their child's burn. Measures were the PCL-5, the Impact of Event Scale-Revised (IES-R), the Montgomery–Åsberg Depression Rating Scale (MADRS), and the Perceived Stress Scale (PSS). Burn severity of the child and sociodemographic variables was obtained.

**Results:**

The parents’ average PCL-5 scores were low to moderate. The internal consistency of the PCL-5 was satisfactory, with Cronbach's alpha ranging from 0.56 to 0.77 and mean inter-item correlations ranging from 0.22 to 0.73 for the four PCL-5 subscales and the PCL-5 total. The PCL-5 subscales were moderately to highly correlated with the corresponding IES-R subscales as well as MADRS and PSS (*p<*0.05), whereas associations with sociodemographics and burn severity were low to moderate.

**Conclusions:**

This study provides preliminary support for the use of PCL-5. The results indicate satisfactory psychometric properties of the PCL-5 as measured with internal consistency, test–retest reliability, and aspects of convergent validity.

A child burn is a very stressful experience for the parents as well as for the child. Symptoms of anxiety, depression, and posttraumatic stress disorder (PTSD) have been frequently reported in parents following a child's burn (Bakker, Maertens, Van Son, & Van Loey, 2013). However, with the recent revisions of the diagnosis of PTSD there is a lack of validated diagnostic instruments to properly assess these symptoms. A majority of parents have acute stress reactions during the first months after the burn (Bakker, Maertens, et al., 2013; Hall et al., 2006), and symptoms of posttraumatic stress have been reported in 14–42% of parents up to 5 years after the burn (Bakker, Maertens, et al., 2013). One study found that 16% of the parents fulfilled the criteria for PTSD up to 7 years post-burn (Rizzone, Stoddard, Murphy, & Kruger, 1994).

Proposed risk factors for parental PTSD symptoms following pediatric burn injury include burn severity (Hall et al., 2006; Rizzone et al., 1994), being a mother (Bakker, Van der Heijden, Van Son, & Van Loey, 2013), and a younger age of the child at the time of injury (Odar et al., 2013).

The PTSD diagnosis has recently undergone substantial revision in the fifth edition of the Diagnostic and Statistical Manual of Mental Disorders (DSM-5, American Psychiatric Association, 2013). The diagnosis now comprises 20 symptoms instead of the previous 17, grouped into four symptom clusters: Intrusion, Avoidance, Negative alterations of cognitions and mood, and Alterations in arousal and reactivity.

An implication of these revisions is that instruments assessing symptoms of PTSD need to be revised according to the DSM-5. The PTSD Checklist (PCL), which is one of the most widely used self-report instruments for the assessment of PTSD symptoms, now exists in a modified fifth version with 20 items, each one corresponding to a single symptom of PTSD (PTSD Checklist for DSM-5, or PCL-5) (Weathers et al., 2013). It is one of the most widely used measures for PTSD in both research and clinical settings with satisfactory psychometric properties (McDonald & Calhoun, 2010).

To date there is a lack of studies assessing the psychometric properties of the PCL-5 in its Swedish translation. The aim of the present study was to examine the psychometric properties of the PCL-5 in parents of children with burns. Reliability will be assessed by internal consistency and test–retest. Aspects of validity will be assessed with intercorrelations of the PCL-5 and correlations with measures of traumatic stress, general stress, and depression, as well as burn severity and sociodemographic variables. It is hypothesized that the PCL-5 total score and subscale scores will be intelligibly and positively associated with measures of traumatic stress covering intrusion, avoidance, hyperarousal, depression, and general stress.

## Methods

### Participants and procedure

The participants included in the present study are individuals taking part in a randomized controlled trial of a self-help and information program for parents of children with burns (Sveen et al., 2015). The Uppsala Burn Center and the Linköping Burn Center are the two main Swedish burn centers with nationwide responsibility for treating patients with severe burns. Admission criteria are based on the recommendations of the American Burn Association. The sample for this study comprised of consecutively admitted patients at the two burn centers between January 2009 and December 2013. Inclusion criteria for the parents were: (1) age of the child <18 years at the time of study, (2) not being treated for burn in conjunction with the child, (3) the burn of the child was unintentional and no suspicion of abuse or neglect of the child as a cause of burn, and (4) ability to understand and respond in Swedish. Parents of 215 children fulfilled the inclusion criteria for the intervention study and were invited by an information letter including a consent form. Non-responders received a telephone call. Of the 215 eligible families, 115 could not be reached and 30 declined, thus 70 families (104 parents or step-parents) agreed to participate, and 62 of these parents completed the assessment.

The data were collected online using a secure web portal as part of a pre-assessment for the intervention study. Data collection took place before randomization and data from the control group's second assessment 6 weeks after the pre-assessment were used to assess test–retest reliability (*n=*27). The study was approved by the Regional Ethics Review Board in Uppsala.

### Measures

#### The PTSD Checklist for DSM-5

The PCL-5 was used to assess symptoms of PTSD (Weathers et al., 2013). It contains 20 items that can be divided into four subscales corresponding to the clusters B–E in the DSM-5: Intrusion (five items), Avoidance (two items), Negative alterations in cognitions and mood (seven items), and Alterations in arousal and reactivity (six items). The items are rated on a 5-point Likert-type scale (0=“not at all” to 4=“extremely”). The items refer to the past month pertaining to a specific event, that is, the burn injury. Total scores range from 0 to 80 and a preliminary cutoff score of 38 is recommended as indicating PTSD caseness (Weathers et al., 2013). The PCL-5 was translated into Swedish by researchers at the National Centre of Disaster Psychiatry at Uppsala University. It was subsequently back-translated by a professional translator and approved by the original authors.

#### The Impact of Event Scale-Revised

The widely used Impact of Event Scale-Revised (IES-R; Weiss & Marmar, 1997) was employed to assess symptoms of traumatic stress during the past week. It contains 22 items divided into three subscales: Intrusion (eight items), Avoidance (eight items), and Hyperarousal (seven items). The items are rated on a 4-point Likert-type scale: 0, 1, 3, and 5, where 0 equals no symptom and 5 equals a high frequency of the symptom during the past week pertaining to a specific event, that is, the burn injury. Total scores range from 0 to 110, with a recommended cutoff of 40 for PTSD caseness (Sveen, Low, et al., 2010). The Swedish version of the IES-R has shown excellent psychometric properties in previous studies after burns (Sveen, Low, et al., 2010; Sveen, Orwelius, et al., 2010). The IES-R demonstrated good internal consistency in the current sample: Cronbach's alpha=0.92 and mean inter-item correlations (MIIC)=0.39 for the IES-R total; α=0.84, MIIC=0.43 for the Intrusion subscale; α=0.78, MIIC=0.34 for the Avoidance subscale; and α=0.75, MIIC=0.49 for the Hyperarousal subscale.

#### Perceived Stress Scale-14 items

The Perceived Stress Scale-14 items (PSS-14; Cohen, Kamarck, & Mermelstein, 1983) was used to measure perceived stress in daily life during the past month. It consists of 14 items rated on a 5-point Likert-type scale (0=never, 4=very often). Total score ranges from 0 to 56 (α=0.84, MIIC=0.29).

#### The Montgomery–Åsberg Depression Rating Scale

The Montgomery–Åsberg Depression Rating Scale (MADRS; Montgomery & Asberg, 1979) was used to measure symptoms of depression during the past 3 days. It consists of nine items rated on a scale from 0 to 6. A higher score reflects more symptoms of depression. Total score ranges from 0 to 54 (α=0.87, MIIC=0.47).

#### Injury, child, and parent characteristics

Data regarding length of stay as inpatients at the burn center (LOS), total body surface area burned (TBSA burned), TBSA with full-thickness burns (TBSA-FT), age, and gender of the child were gathered from the children's medical records. The following parent characteristics were obtained in the questionnaire: age, gender, marital status (0=single, 1=married/co-habiting), working status (0=unemployed/parental leave, 1=working/studying), and education divided into low/medium (12 years’ compulsory school or high school degree/upper secondary school), and high (university degree).

### Statistical analyses

All analyses were performed with the statistical package IBM^®^ SPSS^®^ Statistical package version 21. The internal consistency of the PCL-5 was evaluated with Cronbach's alpha coefficients and MIIC. A Cronbach's alpha of 0.70 and above is regarded as satisfactory (Nunnally, 1978), while the recommended range of MIIC is 0.15–0.50 (Clark & Watson, 1995). The MIIC is a “straightforward” test of internal consistency and is not affected by the number of items in a given subscale as opposed to Cronbachs's alpha. Test–retest reliability and aspects of validity were assessed with Spearman's rho correlations. In order to increase comparability among the different measures, means and medians are presented in addition to the summated scores that are used in clinical practice. Non-parametric analyses were used due to the restricted sample size and the distributions of scores on the PCL-5.

## Results

### Injury, child, and parent characteristics

Sixty-two parents (42 mothers and 20 fathers, 13 of whom were parents of the same child) participated in the study. The mean age of the parents was 37.3 years (SD=6.1, range 23–50 years), 92% were married or cohabitant, 94% were working or studying, and 47% had a university degree.

There were 49 children (22 girls and 27 boys) of the 62 parents. Cause of burn was scalding (*n*=37), contact burns (*n*=7), explosion (*n*=2), flame (*n*=2), and chemical (*n*=1). Demographics and burn characteristics of the children are summarized in Table 1.

**Table 1:** Characteristics of the child (*n=*49)

	Mean	SD	Range
Age of child at injury (years)	2.9	3.6	0.1–15.0
Age of child at study (years)	5.8	3.6	0.9–18.0
Time since injury (years)	2.9	1.3	0.8–5.6
TBSA (%)	9.2	7.0	1.2–30.5
TBSA-FT (%)	2.1	4.3	0–21.5
Length of stay in hospital (days)	7.3	7.8	1–36

TBSA = total body surface area burned, TBSA-FT = total body surface area full thickness burns.

### Scoring distribution

The scoring distribution for the PCL-5 subscales and total score, as well as for each item, is presented in Table 2. Scores for the other measurements are presented in Table 3. The PCL-5 subscale mean scores were low to moderate, and the mean total score was 5.5. No parent scored above the preliminary cutoff score of 38 for PTSD caseness. However, three individuals scored above the cutoff for PTSD caseness on the IES-R.

**Table 2:** Score distribution and reliability for the PCL-5 (*n* = 62)

	Items (*n*)	Mean (SD)	Median (range)	Cronbach's alpha	MIIC	Test–retest*
PCL-5 subscales						
Intrusion	5	0.9 (1.3)	0.0 (0–6)	0.57	0.23	0.58**
Avoidance	2	0.5 (1.1)	0.0 (0–6)	0.74	0.73	0.49**
Cognition and mood	7	2.5 (3.2)	1.0 (0–14)	0.78	0.34	0.63***
Arousal and reactivity	6	1.7 (2.5)	0.0 (0–9)	0.77	0.40	0.77***
PCL-5 total	20	5.5 (7.1)	2.0 (0–28)	0.90	0.32	0.66***
Item 1		0.52 (0.70)	0.0 (0–3)			
Item 2		0.03 (0.18)	0.0 (0–1)			
Item 3		0.10 (0.43)	0.0 (0–3)			
Item 4		0.18 (0.30)	0.0 (0–1)			
Item 5		0.10 (0.35)	0.0 (0–2)			
Item 6		0.35 (0.77)	0.0 (0–4)			
Item 7		0.13 (0.38)	0.0 (0–2)			
Item 8		0.34 (0.65)	0.0 (0–3)			
Item 9		0.31 (0.74)	0.0 (0–3)			
Item 10		0.60 (0.91)	0.0 (0–4)			
Item 11		0.47 (0.78)	0.0 (0–3)			
Item 12		0.19 (0.51)	0.0 (0–2)			
Item 13		0.32 (0.65)	0.0 (0–3)			
Item 14		0.23 (0.56)	0.0 (0–3)			
Item 15		0.23 (0.46)	0.0 (0–2)			
Item 16		0.08 (0.34)	0.0 (0–2)			
Item 17		0.45 (0.86)	0.0 (0–4)			
Item 18		0.31 (0.53)	0.0 (0–2)			
Item 19		0.26 (0.54)	0.0 (0–2)			
Item 20		0.34 (0.75)	0.0 (0–4)			
		Mothers (*n* = 42)		Fathers (*n* = 20)		
		Mean (SD)		Mean (SD)	*p-value*	
Intrusion		1.0 (1.3)		0.7 (1.2)	*ns*	
Avoidance		0.5 (0.9)		0.6 (1.4)	*ns*	
Cognition and mood		2.5 (3.0)		2.3 (3.7)	*ns*	
Arousal and reactivity		1.8 (2.6)		1.5 (2.1)	*ns*	
PCL-5 total		5.8 (7.0)		4.9 (7.3)	*ns*	

MIIC = mean inter-item correlations.

* Retest: n=27

** *P*<0.01

*** *P*<0.001.

**Table 3:** Scoring distribution for IES-R, MADRS, and PSS (*n* = 62)

	Mean (SD)	Median (range)
IES-R total	14.6 (14.6)	10.0 (0–75)
IES-R subscales		
Intrusion	7.3 (6.7)	5.5 (0–34)
Avoidance	4.7 (5.4)	3.0 (0–25)
Hyperarousal	2.6 (3.9)	1.0 (0–16)
MADRS	4.6 (5.4)	3.0 (0–25)
PSS	20.5 (7.8)	19.0 (5–41)

IES-R=the Impact of Event Scale Revised, MADRS=The Montgomery–Åsberg Depression Rating Scale, PSS=Perceived Stress Scale.

### Internal consistency

The Cronbach's alpha scores were acceptable to good (α range=0.57–0.78) for the subscales and high (α=0.90) for the total scale. The MIIC were good and ranged between 0.23 and 0.40, except for the avoidance subscale with a MIIC of 0.73. Test–retest coefficients were significant and ranged from 0.49 to 0.77 (Table 2).

### Validity (Table 4)

#### PCL-5 intercorrelations

The PCL-5 subscale intercorrelations ranged between 0.45 and 0.76, which indicates that the subscales tap into a similar construct but are not interchangeable.

**Table 4:** PCL-5 Intercorrelations and correlations with IES-R, MADRS, PSS, burn severity, and sociodemographic characteristics (Spearman's rho)

	PCL-5				
	
	Intrusion	Avoidance	Cognition and mood	Arousal and reactivity	PCL total
PCL-5					
Intrusion	1				
Avoidance	0.52**	1			
Cognitions and mood	0.76**	0.45**	1		
Arousal and reactivity	0.65**	0.50**	0.72**	1	
PCL-5 total	0.84**	0.57**	0.93**	0.85**	1
IES-R					
Intrusion	0.53**	0.37**^a,^ ^b^	0.48**	0.39**^d,^ ^e^	0.48**^f^
Avoidance	0.52**	0.55**^b,^ ^c^	0.51**	0.47**	0.57**
Hyperarousal	0.44**	0.34**^c^	0.41**	0.62**^e^	0.50**
IES-R total	0.55**	0.46**^a^	0.54**	0.53**^d^	0.58**^f^
MADRS total	0.47**	0.47**	0.60**	0.52**	0.60**
PSS total	0.47**	0.41**	0.51**	0.61**	0.56**
Child burn severity					
TBSA Total	0.21	0.01	0.27*	0.09	0.24
TBSA-FT	0.14	−0.17	0.10	0.16	0.12
LOS	0.21	−0.13	0.19	0.10	0.21
Time since injury	−0.22	−0.20	−0.15	−0.10	−0.18
Sociodemographics					
Child age at injury	−0.01	0.00	0.15	0.08	0.15
Child age at study	−0.11	−0.13	–0.05	−0.06	−0.04
Child gender	−0.22	−0.19	−0.35**	−0.28*	−0.38**
Parent's age	0.04	−0.04	−0.02	0.06	0.04
Parent's gender	−0.19	0.04	−0.14	−0.05	−0.10
Educational level	0.07	0.05	0.17	0.02	0.09
Working status	−0.14	0.02	−0.07	−0.19	−0.10

PCL-5=The PTSD Checklist 5th version, IES-R=the Impact of Event Scale Revised, MADRS=The Montgomery–Åsberg Depression Rating Scale, PSS=Perceived Stress Scale, TBSA=total body surface area burned, TBSA-FT=total body surface area full thickness burns, LOS=Length Of Stay in the burn center, Gender: 1=male, 0=female, Educational level: 1=higher, 0=low/moderate, Working status: 1=Working/studying, 0=unemployed/parental leave.

* *p*<0.05

** *p*<0.01. Same lowercase letters within each PCL-5 subscale represent statistically significant differences in correlations (*P*<0.05).

#### PCL-5 total score

There were significant correlations between the PCL-5 total score and the IES-R total and IES-R subscales (range of *rho=*0.48–0.58), the strongest correlation was with the IES-R total. The PCL-5 total correlated with MADRS (*rho=*0.60) and PSS (*rho=*0.56). There was a positive significant association with female gender of the child (*rho=*0.38).

#### PCL-5 intrusion subscale

There were significant correlations between the Intrusion subscale and the IES-R total and all three IES-R subscales (range of *rho=*0.44–0.51), MADRS (*rho=*0.47), and PSS (*rho=*0.47).

#### PCL-5 avoidance subscale

There were significant correlations between PCL-5 Avoidance subscale and the IES-R total score and all three subscales (range of *rho=*0.34–0.55). The strongest correlation was, as expected, with the IES-R Avoidance subscale, which was significantly stronger than the correlations with the IES-R Intrusion and Hyperarousal subscales. The Avoidance subscale also correlated with MADRS (*rho=*0.47) and PSS (*rho=*0.41).

#### PCL-5 negative alterations in cognitions and mood subscale

There were significant correlations between the subscale and the IES-R total score and all three subscales (*rho=*0.41–0.54). The strongest association was, as hypothesized, with MADRS (*rho=*0.60). This subscale also correlated with PSS (*rho=*0.51) and TBSA total (*rho=*0.27). There was a positive significant correlation with female gender of the child (*rho=*0.35).

#### PCL-5 alterations in arousal and reactivity subscale

This subscale correlated with the IES-R total score and all three subscales; the strongest correlation was, as expected, with the IES-R Hyperarousal subscale and it was significantly higher than with the IES-R Intrusion. It was also significantly correlated with the PSS and the MADRS, and there was a positive significant correlation with female gender of the child (*rho=*0.28).

Due to potential dependency of data, all analyses were repeated while excluding one of the parents in each of the participating 13 pairs (who were parents of the same child); however, there were no substantial differences to the results.

## Discussion

This pilot study provides preliminary support for the use of PCL-5. The analyses of reliability and validity indicated that the PCL-5 has satisfactory psychometric properties in parents of children with burns. The overall internal consistency of the PCL-5 was satisfactory as assessed with Cronbach's alpha values and MIIC. However, the Intrusion subscale had a moderate Cronbach's alpha value of 0.57, and a careful scrutiny revealed that the internal consistency did not improve by excluding any item. In contrast, the Cronbach's alpha value for the IES-R Intrusion subscale was high (α=0.84). A possible explanation is that the two subscales in part consist of different items, that is, the Intrusion item regarding physiological reactions when reminded of the event belongs to the Hyperarousal subscale in the IES-R, and the Intrusion subscale of the IES-R contains one item concerning sleep problems, which is not included in the PCL-5. The Avoidance subscale had satisfactory alpha (0.74) but a MIIC (0.73) above the recommended cutoff, indicating a substantial overlap between the two items.

The results indicate that the PCL-5 has convergent validity. PCL-5 intercorrelations were moderate to high, which suggests that the items tap into a similar construct but are not interchangeable. Furthermore, the PCL-5 total and subscales were highly correlated with the IES-R total and subscales as well as with MADRS and PSS, which is in agreement with the hypotheses of the study. There are several overlapping symptoms between depression and PTSD, and furthermore these two conditions are commonly comorbid (Brady, Killeen, Brewerton, & Lucerini, 2000).

This investigation indicates that aspects of discriminant validity of the PCL-5 are low, for instance, that PCL-5 correlation with IES-R total score was not significantly stronger than its association with MADRS or PSS. This result could be due to the rather low symptom level in the sample, and maybe in a more symptomatic sample, the discriminant validity would be better.

The PCL-5 was not associated with suggested risk factors for PTSD such as female gender of the parent, low educational level, young age of child, or burn severity. The only significant association found was between burn size (total TBSA burned) and Negative alterations in cognitions and mood, and the association was weak. One recent study on minor burns in children found an association between parental PTSD symptoms and young child age at time of injury, but no associations with parent's age, educational level, or child's burn severity (Odar et al., 2013). These findings are somewhat similar to those in our study, although we included children with minor to moderate burns. These associations may differ in samples with more severe burns, or with families with more diverse sociodemographic backgrounds. In the present study, there was a positive association between female gender of the child and higher scores on the PCL-5 total and the subscales Negative alterations in cognitions and mood, and Alterations in arousal and reactivity, which has not been found in previous studies in parents of children with burns (De Young, Hendrikz, Kenardy, Cobham, & Kimble, 2014; Willebrand & Sveen, 2016).

At present, other studies have reported similar Cronbach's alpha values for PCL-5, ranging from 0.76 to 0.97 (Armour et al., 2015; Frewen, Brown, Steuwe, & Lanius, 2015; Hoge, Riviere, Wilk, Herrell, & Weathers, 2014; Keane et al., 2014), which are comparable with our study (α=0.90). However, there are no studies on other aspects of reliability and validity to compare with at this stage.

The limitations of the present study are the small sample size and the low symptom level of the sample. Conclusions should therefore be regarded as preliminary. Another limitation is that the instrument was not validated against a structured interview for PTSD. Strength of the study is the concurrent validation against well-used and validated measures of traumatic stress, that is, the IES-R, as well as measures of general stress and depression. Another strong point is that there were no missing responses on any of the measures, that is, there was no internal attrition and the raw data held a high quality.

A preliminary conclusion is that the PCL-5 has satisfactory psychometric properties, as measured with internal consistency, test–retest reliability, and aspects of convergent validity. The discriminant validity was not strong; however, this might be a reflection of the overlap in symptoms between depression, general stress, and PTSD. Thus, the overall positive result motivates further studies with larger samples and preferably with participants who have larger variation in symptoms levels, to replicate the findings in this study and to expand the knowledge regarding the validity of the PCL-5.
